# The prevalence of cardiac and hepatic iron overload in patients with kidney failure: A protocol for systematic review and meta‐analysis

**DOI:** 10.1002/hsr2.692

**Published:** 2022-06-08

**Authors:** Abdulqadir J. Nashwan, Mohamed A. Yassin, Alaa Abd‐Alrazaq, Farag Shuweihdi, Hanan F. Abdul Rahim, Mujahed Shraim

**Affiliations:** ^1^ Department of Nursing, Hazm Mebaireek General Hospital Hamad Medical Corporation Doha Qatar; ^2^ Department of Public Health, College of Health Sciences, QU Health Qatar University Doha Qatar; ^3^ Hematology and Oncology, Hamad General Hospital Hamad Medical Corporation Doha Qatar; ^4^ AI Center for Precision Health Weill Cornell Medicine‐Qatar Doha Qatar; ^5^ School of Medicine, Leeds Institute of Health Sciences University of Leeds Leeds UK

**Keywords:** end‐stage renal disease, hemochromatosis, iron overload, kidney failure, meta‐analysis, systematic review

## Abstract

**Introduction:**

Few studies have reported cardiac and hepatic iron overload in patients with kidney failure or end‐stage renal disease and the current evidence regarding the prevalence is still scarce. To the best of the authors' knowledge and following an exhaustive search; no systematic review/meta‐analysis has estimated the aggregated prevalence of cardiac and hepatic iron overload in this patient population.

**Aim:**

This review aims to estimate the prevalence of hepatic and/or cardiac iron overload in patients with kidney failure who are receiving hemodialysis, peritoneal dialysis, or underwent kidney transplants.

**Methods:**

A systematic review with meta‐analysis will be conducted and reported in line with PRISMA guidelines. MEDLINE and Embase bibliographic databases will be searched using a comprehensive list of controlled vocabularies and keywords to identify relevant studies. All studies reporting the prevalence of hepatic and/or cardiac iron overload prevalence in patients with kidney failure will be considered. Risk of bias assessment for included studies will be conducted based on the study design. StataBE v17 and MetaXL v5.3 will be utilized to perform the meta‐analysis.

**Discussion:**

The findings of this systematic review and analysis are expected to give information on the prevalence of iron overload among patients with kidney failure, which will optimize interventions and guide future research in this understudied field.

## 1 INTRODUCTION

Chronic kidney disease (CKD) is a challenging health issue across the world. Globally, the prevalence estimates for CKD range between 11.7% and 15.1%.^1^ Moreover, the development of CKD to cardiovascular diseases and kidney failure has a direct influence on morbidity and death rates.^2^ About 1 in 7 adults in the United States (around 37 million people) are affected by CKD, which is more often caused by chronic conditions such as hypertension and diabetes.^3^ CKD diagnosis is made if the patient has a glomerular filtration rate (GFR) of <60 ml/min per 1.73 m^2^, increased urinary albumin excretion, or both, for at least 3 months, irrespective of the underlying cause and kidney failure is the 5th stage of CKD (GFR < 15 ml/min/1.73 m^2^).^4^, ^5^ While anemia represents the feature complication in patients with kidney failure,^6^ the possibility of iron overload toxicity associated with continuous intravenous (IV) iron replacement is currently one of the most contentious issues in the management of anemia in patients with kidney failure.^7^

Iron excess may have a negative impact on the heart (e.g., heart failure, arrhythmias, and sudden cardiac death), the liver (hepatocellular carcinoma), and causes other complications such as diabetes mellitus, hypogonadism, and musculoskeletal and skin‐related conditions.^8^, ^9^ In addition, higher iron stores may negatively affect the immune‐regulatory balance, weakening the immune system and hindering effective treatment of underlying illnesses.^10^ Unfortunately, the low creatinine clearance of most iron chelators, limits the options for treating iron excess in kidney failure patients who undergo hemodialysis, peritoneal dialysis, or kidney transplant.^11^ This creates a clinical problem in terms of balancing the need to correct hemoglobin while avoiding iron excess.^12^

The prevalence of hepatic and cardiac iron overload in kidney failure patients was reported by few studies and limited to the cases of hepatic iron overload in hemodialysis patients which ranges roughly between 50% and 100%.^13^, ^14^ However, an exhaustive search revealed no systematic review/meta‐analysis has been conducted to synthesis the epidemiologic evidence on the prevalence of hepatic and cardiac iron overload in kidney failure patients. Thus, this systematic review and meta‐analysis aims at estimating the prevalence of iron overload (cardiac and/or hepatic) in patients who receive hemodialysis, peritoneal dialysis, or underwent kidney transplants.

## 2 METHODS

This procedure for a systematic review and meta‐analysis was developed in accordance with PRISMA‐P^15^ reporting guidelines. The protocol has been registered at PROSPERO and can be found with the registration number: CRD42022306803.

### 2.1 Search strategy

An extensive search will be conducted for published studies with no time restrictions. The bibliometric databases MEDLINE and Embase will be searched using a complete set of controlled vocabularies, including medical subject heading (MeSH) and Emtree keywords, as well as free‐text terms pertaining to iron overload/toxicity and CKD. For example, MEDLINE will be searched using the following terms: (“chronic kidney diseases” OR “chronic renal diseases” OR “chronic kidney insufficiency” OR “chronic renal insufficiency” OR “end stage renal disease” OR “hemodialysis” OR “peritoneal dialysis” OR “kidney transplant*” OR “renal transplant*”) AND (“iron imbalance” OR “iron overload” OR “iron deposition” OR “iron toxicity” OR “haemochromatosis” OR “hemochromatosis”) AND (“heart” OR “cardiac” OR “Liver” OR “hepatic”). In addition, a backward and forward reference checking will be performed to identify further eligible studies. In addition, the citations of included studies will be traced using the Web of Science and Scopus citation indices to identify any relevant studies. The search terms will be piloted and refined as needed.

### 2.2 Study selection criteria

All epidemiologic studies reporting on the prevalence of hepatic and/or cardiac iron overload (quantified by magnetic resonance imaging [MRI]) among adults with 18 years and above and with a confirmed diagnosis of kidney failure receiving peritoneal dialysis, hemodialysis, or underwent kidney transplant will be included regardless of the publication's language. Studies about patients aged <18 years or having other co‐existing hematologic or hepatic disorders will be excluded.

All literature, including cross‐sectional, retrospective, prospective cohort studies, and randomized controlled trials (the intervention group) reported in any language (professional English translation will be provided as appropriate) with no time restrictions, will be included. On the other hand, reviews, commentaries, protocols, abstracts, case reports/series, and posters will be excluded.

### 2.3 Screening and data extraction

Studies retrieved from all databases will be exported to EndNote™ to eliminate duplicates. Then, titles, abstracts, and keywords of the remaining publications will be screened to check their eligibility. Lastly, full texts of publications included from the previous step will be checked for eligibility. The following items will be retrieved from the studies: the surname of the first author, publication year, location, setting, gender, design, age of patients, sample size, and the operational definition, prevalence, and severity of hepatic and/or cardiac iron overload (quantified by MRI). In case of missing data or unavailability of the full text, the author/s will be contacted via e‐mail for further clarifications. Two reviewers will independently perform study selection and data extraction. Any discrepancies will be settled by consensus or arbitration by a 3rd reviewer.

### 2.4 Quality assessment and risk of bias

The Newcastle‐Ottawa Scale (NOS)^16^ will be utilized to evaluate the methodologic aspects of observational studies (cross‐sectional, case‐control, and cohort studies), which consists of 8 items evaluating; selection, comparability, and exposure. In addition, the Cochrane Collaboration's Risk of Bias (ROB) Tool will be utilized to evaluate the quality of randomized controlled trials.^17^ Two reviewers will independently appraise the methodological quality of the included studies, and any disagreements will be resolved by consensus or reconciled by a third reviewer. Furthermore, the Schmidt–Hunter method will be utilized to identify and reduce the risk of publication bias.^18^

### 2.5 Statistical analysis

The outcome of interest will be the proportion of patients with kidney failure who have liver and/or cardiac iron overload. For those studies provided; multi‐category prevalence analysis^19^ will be utilized and the estimates will be pooled to arrive at MRI severity‐related prevalence estimate, these proportions will be synthesized based on pre‐defined multi‐groups to improve homogeneity by categorizing into MRI hepatic and cardiac quantification: (1) cardiac: normal (T2* > 20 ms), mild to moderate (T2* = 10–20 ms), and severe (T2* < 10 ms). (2) hepatic: severe (T2* < 1.8 ms), mild to moderate (T2* = 1.8–11.4 ms), and normal (T2* > 11.4 ms).^20^

The pooled effect size and corresponding 95% confidence interval (CI) will be based on double arcsine transformation to stabilize variances when the proportions become closer to 0 or 1.^19^ The Cochran's *Q χ* ^2^ statistic will be utilized to test the statistical heterogeneity of the included studies; and *I* ^2^ will be computed to describe the proportion of total variation due to heterogeneity.^21^ The quality effects model will be utilized to deal with the statistical heterogeneity by yielding a quantitative summary of the pooled effect size as this methodology gives more weight to high‐quality versus low‐quality studies.^22^ Sensitivity analyses will be used to determine the robustness of this meta‐analysis by altering the selection criteria of each study. StataBE v17 and MetaXL v5.3 (www.epigear.com) will be utilized to perform the meta‐analyses.^19^

## 3 DISCUSSION

Iron overload was once thought to be uncommon in hemodialysis patients, but it is currently becoming a more common clinical condition. Recent quantitative MRI studies clearly imply a relationship between IV iron dosage and the risk of iron overload, bringing existing biomarker cutoffs and clinical recommendations into doubt, particularly regarding recommended iron doses.^23^, ^24^

Furthermore, several recent observational studies have revealed that high IV iron levels may increase cardiovascular events and overall mortality in kidney failure patients on hemodialysis.^25^, ^26^, ^27^, ^28^ This newly identified unfavorable impact of IV iron has resulted in significant and constant revisions in the idea and practical approach to IV iron replacement in dialysis patients.

Despite the recent progress in understanding the iron overload (cardiac and hepatic) in patients with hemoglobinopathies,^29^, ^30^, ^31^, ^32^ still, many aspects are unanswered concerning patients with kidney failure. A systematic review and meta‐analysis published in 2019 estimated that the overall prevalence of cardiac iron overload—for example—in patients with thalassemia major was 25% (95% CI: 22%–28%).^33^ However, the percentage is still unknown for patients with kidney failure. This review will estimate the pooled iron overload prevalence (cardiac and/or hepatic) in patients with kidney failure. It will also be expected to provide useful directions for future research in this understudied field. Potential limitations are expected which mainly related to (1) the search may not identify all relevant literature; this limitation will be overcame by implementing a comprehensive search strategy. (2) The potential influence of confounding factors such as demographics, nutritional, dietary intake, and socioeconomic factors and this will be minimized and adjusted while conducting the meta‐analysis part.

## AUTHOR CONTRIBUTIONS

**Abdulqadir J. Nashwan**: conceptualization; methodology; writing–original draft; writing–review & editing. **Mohamed A. Yassin**: methodology; writing–original draft; writing – review & editing. **Alaa Abd‐Alrazaq**: methodology; writing–original draft; writing–review & editing. **Farag Shuweihdi**: methodology; writing–original draft; writing–review & editing. **Hanan F. Abdul Rahim**: methodology; writing–original draft; writing–review & editing. **Mujahed Shraim**: methodology; writing–original draft; writing–review & editing. All authors have read and approved the final version of the manuscript.

## CONFLICT OF INTEREST

The authors declare no conflict of interest.

## TRANSPARENCY STATEMENT

Abdulqadir J. Nashwan affirms that this manuscript is an honest, accurate, and transparent account of the study being reported; that no important aspects of the study have been omitted; and that any discrepancies from the study as planned (and, if relevant, registered) have been explained.
